# Clinical Evaluation of a Fully-automatic Segmentation Method for Longitudinal Brain Tumor Volumetry

**DOI:** 10.1038/srep23376

**Published:** 2016-03-22

**Authors:** Raphael Meier, Urspeter Knecht, Tina Loosli, Stefan Bauer, Johannes Slotboom, Roland Wiest, Mauricio Reyes

**Affiliations:** 1 Institute for Surgical Technology & Biomechanics, University of Bern, Bern, Switzerland; 2 Support Center for Advanced Neuroimaging – Institute for Diagnostic and Interventional Neuroradiology, University Hospital and University of Bern, Bern, Switzerland

## Abstract

Information about the size of a tumor and its temporal evolution is needed for diagnosis as well as treatment of brain tumor patients. The aim of the study was to investigate the potential of a fully-automatic segmentation method, called BraTumIA, for longitudinal brain tumor volumetry by comparing the automatically estimated volumes with ground truth data acquired via manual segmentation. Longitudinal Magnetic Resonance (MR) Imaging data of 14 patients with newly diagnosed glioblastoma encompassing 64 MR acquisitions, ranging from preoperative up to 12 month follow-up images, was analysed. Manual segmentation was performed by two human raters. Strong correlations (*R* = 0.83–0.96, *p* < 0.001) were observed between volumetric estimates of BraTumIA and of each of the human raters for the contrast-enhancing (CET) and non-enhancing *T*_2_-hyperintense tumor compartments (NCE-*T*_2_). A quantitative analysis of the inter-rater disagreement showed that the disagreement between BraTumIA and each of the human raters was comparable to the disagreement between the human raters. In summary, BraTumIA generated volumetric trend curves of contrast-enhancing and non-enhancing *T*_2_-hyperintense tumor compartments comparable to estimates of human raters. These findings suggest the potential of automated longitudinal tumor segmentation to substitute manual volumetric follow-up of contrast-enhancing and non-enhancing *T*_2_-hyperintense tumor compartments.

The accurate and reproducible measurement of tumor size and its changes over time is of crucial importance for diagnosis, treatment planning as well as monitoring of response to oncologic therapy for brain tumors. Current clinical guidelines (RANO/AvaGlio^1^) for response assessment of high-grade glioma patients rely on bidimensional measures. Compared to tumor volumetry, bidimensional measures show several limitations: Increased measurement variability^2^,^3^,^4^,^5^, sensitivity to imaging quality^6^ and difficulties in assessing irregularly shaped, unmeasurable or satellite lesions^7^. Volumetry of a tumor requires an operator to outline the tumor and to differentiate between the different tumor compartments and peritumoral changes, which in turn requires considerable skill and expertise in tumor diagnostics as well as in handling of the respective software. Consequently, manual tumor volumetry is a time-consuming procedure prone to subjectivity and hence large inter-observer variability^8^,^9^,^10^. Fully-automatic segmentation methods constitute a possible solution to these issues. They perform volumetry in a fraction of the usual amount of time, which can take up to one hour per patient, while eliminating intra-observer and inter-observer variability.

A compartmentalisation of high-grade glioma into necrosis, edema, non-enhancing and enhancing tumor has been found to be associated with response to treatment and patient survival^11^,^12^. Recent studies show that a standardised set of Magnetic Resonance (MR) imaging features of these tumor compartments can be used to stratify patients into different risk groups^13^,^14^. In parallel, automatic methods capable of segmenting a high-grade glioma into its subcompartments have been proposed^15^,^16^,^17^. Such methods rely on imaging information from structural MRI (usually native *T*_1_ weighted (*T*_1_w), *T*_1_w gadolinium enhanced, *T*_2_w and FLAIR sequences) and machine learning techniques for data analysis^10^,^18^.

The majority of studies assessing the potential of computer-assisted segmentation methods for brain tumor volumetry have so far focused on preoperative segmentation. In the study of Porz *et al*.^19^ the segmentation results of an automatic method were compared with manually acquired ground truth data of two expert raters for 25 glioblastoma (GBM) patients. The comparison was performed for the complete tumor (including all four tumor compartments), the tumor core (including necrosis, contrast-enhancing and non-enhancing tumor) and the contrast-enhancing tumor. The study of Steed *et al*.^20^ draws a comparison between automatically and manually segmented patient cases extracted from The Cancer Imaging Archive (TCIA). The authors evaluated their method for the segmentation of the enhancing part of the tumor and the FLAIR hyperintensity volume. Both studies found a good agreement between manual and automatic results. Regarding longitudinal segmentation, Weizman *et al*.^21^ proposed a semi-automatic method capable of subdividing low-grade brain tumors into cystic, solid and enhancing regions and tracking of the volumetric evolution of these subcompartments over time. The authors applied their method to 10 patients, comprising a total of 40 MRI scans, and found it to be accurate when compared to manual segmentations. The study of Liberman *et al*.^22^ is methodologically the closest to the study at hand. The authors evaluated an automatic segmentation method on 59 longitudinal MR scans of 13 patients with recurrent GBM undergoing bevacizumab therapy. The method performs a segmentation of the tumor volume into enhancing tumor volume, peri- and non-peri-tumoral edema. The focus of the study was on improving accuracy in therapy response assessment defined by the MacDonald’s criteria^23^ and manual volumetry. In contrast to the study of Liberman *et al*.^22^, our study was performed explicitly in a prospective setting, uses data of patients with newly diagnosed GBM and provides a comparison between the volumetric trend of tumor compartments as estimated by automatic and manual segmentation.

Motivated by the recent developments, we hypothesise that the volumetric trend of tumor compartments in high-grade glioma as captured by an automatic segmentation method is comparable to the trend estimated by time-consuming manual segmentation.

Following previous work, we employ a fully-automated method^17^,^19^ called BraTumIA (Brain Tumor Image Analysis). The aim of our study is to investigate the potential of BraTumIA for longitudinal brain tumor volumetry by comparing the automatically estimated volumes with ground truth data acquired by manual segmentation.

## Materials and Methods

### Data selection

Manual and automatic segmentations were performed on the longitudinal MRI data of 14 consecutive patients selected from two ongoing, prospective clinical trials in our institution. Imaging data of 64 independent MR acquisitions (each encompassing *T*_1_w, *T*_1_w gadolinium enhanced, *T*_2_w and FLAIR sequences, three to six acquisitions per patient), resulting in a total of 256 different MRI images, was analysed. Only patients with newly diagnosed and histologically confirmed glioblastoma multiforme were eligible for inclusion. The same, standardised MR protocol was performed for all patients. The aim of the clinical trials is to improve the reliability of tumor progression evaluation with MRI and MR Spectroscopy (MRS) before, during and after therapy with neurosurgery, radiotherapy, chemotherapy and/or anti-angiogenic therapy. Seven of the 14 patients received a first line therapy with Bevacizumab in combination with radiotherapy. The main exclusion criteria were incomplete MRI data acquisition, previous cranial neurosurgery, Karnofsky perfomance status lower than 70% and pathological organ function (liver, kidney, impaired hematological function). An overview of the patient data is presented in Table 1. The studies were approved by the Local Research Ethics Commission (Kantonale Ethikkommission Bern) and all methods were carried out in accordance with the approved guidelines. All patients provided written informed consent.

### MR Acquisition

We performed a standardised MR protocol for all patients. All sequences were acquired on a 1.5T MR scanner from Siemens (Siemens Avanto and Siemens Area, Siemens, Erlangen/Germany). For manual and automatic segmentation the following sequences were used: i) 2*D T*_2_w MRI sequence with fluid-attenuated inversion recovery impulse (*T*_2_w *FLAIR*) in axial acquisition, *TE* = 80 *ms, TR* = 8000 *ms, FOV* = 256 × 256 *mm*^2^, *FA* = 120°, anisotropic voxel size of 1 *mm* × 1 *mm* × 3 *mm*; ii) 3*D T*_2_w *SPACE* in sagittal acquisition, *TE* = 380 *ms, TR* = 3000 *ms, FOV* = 256 × 256 *mm*^2^, *FA* = 120°, isotropic voxel size of 1 *mm* × 1 *mm* × 1 *mm*; iii) 3*D T*_1_w MPR without contrast enhancement in sagittal acquisition, *TE* = 2.67 *ms, TR* = 1580 *ms*, FOV = 256 × 256 *mm*^2^, *FA* = 8°, isotropic voxel size of 1 *mm* × 1 *mm* × 1 *mm*; iv) 3*D T*_1_w with gadolinium contrast enhancement in sagittal acquisition, *TE* = 4.57 *ms, TR* = 2070 *ms, FOV* = 256 × 256 *mm*^2^, *FA* = 15°, isotropic voxel size of 1 *mm* × 1 *mm* × 1 *mm*.

### Manual Segmentation

The different MR sequences were skull-stripped^24^ and co-registered on the *T*_1_w gadolinium enhanced sequence using a rigid transformation. This step is part of the BraTumIA software and facilitated the comparison to the automatic segmentations which were generated from the same co-registered images. After that, the images were manually segmented by two human raters blinded to clinical history and diagnosis. The raters used all four MR sequences (*T*_1_w, *T*_1_w gadolinium enhanced, *T*_2_w and FLAIR) simultaneously to annotate a data set. The images were strictly segmented according to their timing, starting with the preoperative image, and independent of the other MR acquisitions of the same patient (i.e. in a prospective fashion). Both raters adhered to a predefined segmentation protocol^25^ and used 3D Slicer^26^ for generating a manual segmentation of the complete tumor into necrosis, edema, non-enhancing and enhancing tumor. Rater-1 is an experienced neuroradiologist with several years of experience in brain tumor image analysis, whereas Rater-2 is a M.D. master student previously trained in neuroimaging with initial experience in the field. In two out of the total 64 MR acquisitions the automatic skull-stripping failed and a manual correction was applied.

### Fully-automatic Segmentation

For the purpose of automatic segmentation we employed the software BraTumIA^15^,^17^. BraTumIA allows the user to load four standard MRI sequences (*T*_1_w, *T*_1_w gadolinium enhanced, *T*_2_w and FLAIR images) that constitute a neurooncological MR protocol according to the RANO criteria^27^. The processing of the imaging information starts with the generation of a brain mask used for automatic skullstripping. This step is followed by a multimodal rigid registration where the *T*_1_w gadolinium enhanced image serves as template (i.e. all images are aligned to this image). Finally, a bias-field correction^28^ is applied and the image intensities are normalised^29^.

After preprocessing, a machine learning-based framework is used for performing segmentation via voxel-wise tissue classification. BraTumIA classifies every voxel into either one of three unaffected tissue classes (gray matter, white matter and cerebrospinal fluid) or into either one of four different tumor classes (necrosis, edema, non-enhancing and enhancing tumor). To perform this classification, the software starts with the extraction of voxel-wise feature vectors composed of appearance-sensitive features (multisequential intensities and intensity differences, first-order and gradient textures) and context-sensitive features (atlas-normalised coordinates, multi-scale symmetry features and ray features)^17^. Based on this feature vector, every voxel is classified by a decision forest^30^,^31^ into one of the tissue classes. The strengths of the decision forest classifier are that it can handle high-dimensional input data (BraTumIA employs a 237-dimensional feature vector), it can handle multi-label classification problems (BraTumIA performs a segmentation into seven different tissue classes) and its output is a probability distribution over the different tissue classes. The predicted tissue class for a particular voxel is chosen to be the one with the highest probability. In a final step, the label map generated by the decision forest is refined by a regularisation, which enforces spatial consistency of classified voxels with respect to their neighborhood through a conditional random field based optimisation^15^.

In contrast to Porz *et al*.,^19^ the training data of 36 preoperative patient cases for BraTumIA was enlarged with longitudinal imaging data sets. For a subset of nine patients, we obtained nine immediate postoperative images. Moreover, for four out of these nine patients we obtained additional nine follow-up images (acquired within one to six months after surgery). This led to a total of 54 imaging data sets of 36 different patients used for training.

### Statistical Analysis

The statistical analysis was targeted at the two morphologically most discriminative tumor compartments, which are the contrast-enhancing tumor (CET) and the non-enhancing *T*_2_-hyperintense part (NCE-*T*_2_) of the tumor following the modified RANO recommendations^1^,^27^. In our analysis, the latter encompasses the segmentation of the non-enhancing tumor and edema. We did not include necrosis due to the fact that this compartment is usually completely resected and reappears often late after surgery showing initially a small volume.

Our statistical analysis followed a descriptive approach. Absolute and relative volumes were plotted against time. Based on the imaging protocol, we defined seven different time points: *t*_*pre*_ (preoperative image), *t*_*post*_ (immediate postoperative image), *t*_1_ (one month follow-up), *t*_3_ (three months follow-up), *t*_6_ (six months follow-up), *t*_9_ (nine months follow-up), *t*_12_ (12 months follow-up). Absolute volumes measured by a particular rater were expressed relative to the preoperative volume 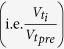. Overlap between segmentation results was evaluated using the Dice-coefficient. Tumor volumes smaller than 90 *mm*^3^ were not considered for computing the Dice-coefficient due to the Dice-Coefficient being overly sensitive for changes in small volumes.

To quantify the agreement between raters regarding tumor volume progression, the slope between consecutive time points was first measured for each rater 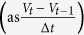. Negative and positive slopes then correspond to shrinking and growing of tumor volumes, respectively. A different sign (negative or positive) between raters is regarded as a disagreement. The total number of disagreements is then used to characterise the disagreement on the complete longitudinal tumor volume progression. Furthermore, we computed the relative increase or decrease of a particular volume measured between two consecutive time points for each rater: 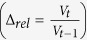. For visualisation, we computed the decimal logarithm of this ratio. We refer to this simply as *disagreement plot*. A stable volume measured from one to another time point would yield a value of zero. A disagreement between raters would result in one or more data points lying on different sides with respect to the zero line. If such a disagreement occurs, we measure the absolute difference (along the y-axis) between the disagreeing raters. This analysis was performed for all possible pairs of raters over the complete data set. The results can then be summarised in what we refer to as a *disagreement matrix*. Every entry of the matrix corresponds to the total sum of all measured disagreements (differences). This yields more information about the extent to which two raters are disagreeing with each other. The matrix is symmetric due to the fact that we measure the absolute difference.

In the remainder of this paper, we refer to BraTumIA (B) as well as the human raters (R1 & R2) simply as *raters* for the sake of clarity, but make a distinction between automated and manual results when appropriate. For the statistical analysis, we used the R software package (R Development Core Team).

## Results

**Figure 1:**
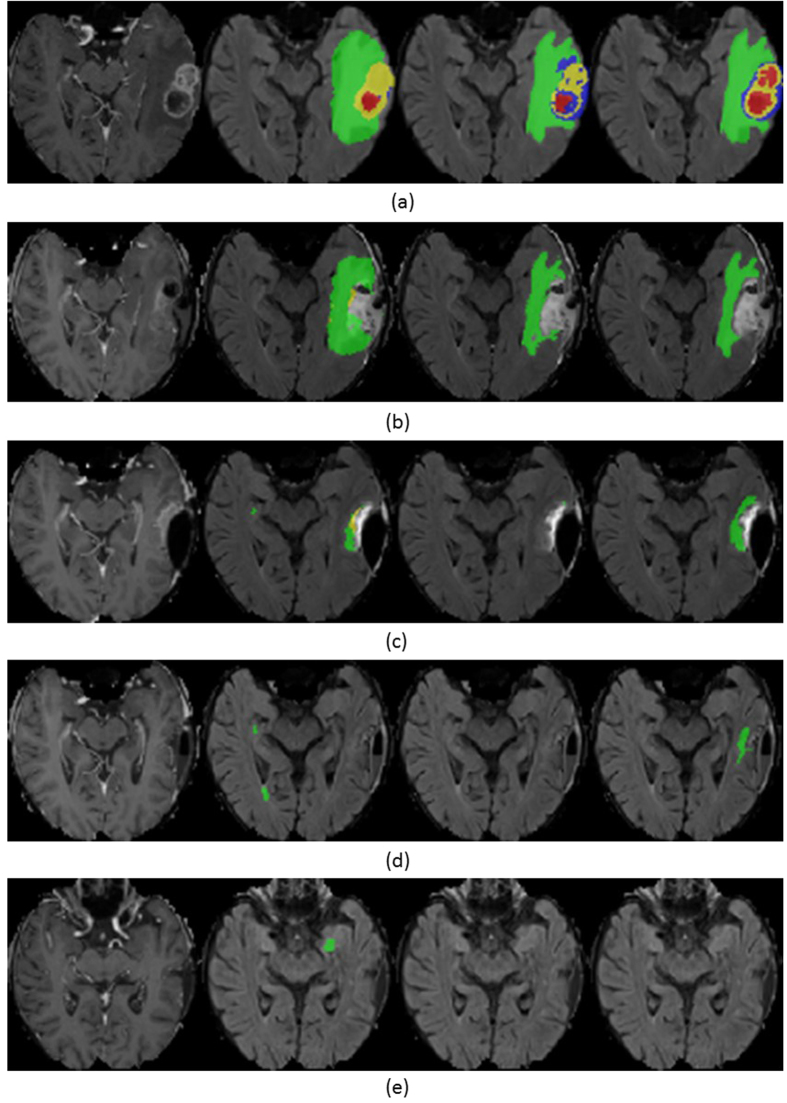
Segmentation of necrosis (red), edema (green), enhancing tumor (yellow) and non-enhancing tumor (blue) in patient “d”. From top to bottom, consecutive time points are shown. From left to right: *T*_1_ post-contrast-weighted image, FLAIR-weighted image overlayed with segmentation generated by BraTumIA, segmentation generated by Rater-1 and segmentation generated by Rater-2.

**Figure 2:**
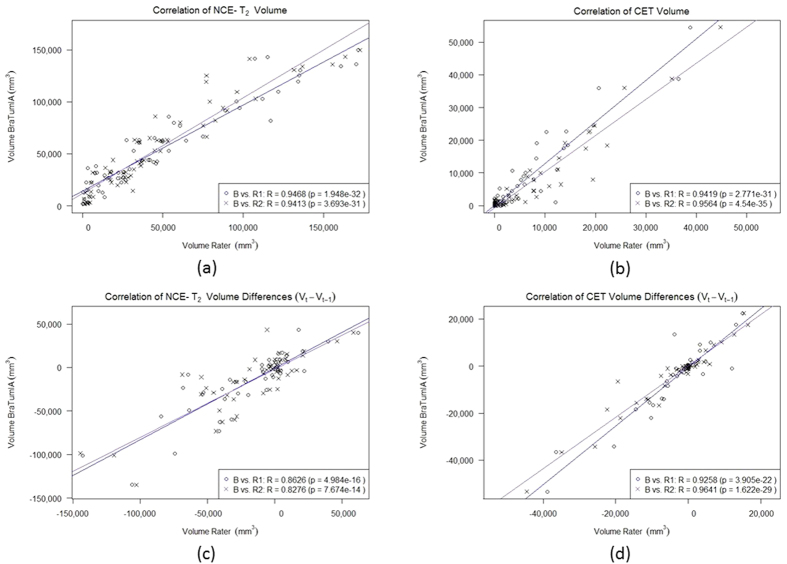
Volumes (*n* = 64) and volume differences (*n* = 50) as measured by BraTumIA are plotted against estimates of Rater-1 (circles, blue line) and of Rater-2 (crosses, violet line).

An exemplary segmentation of a particular patient over time for each BraTumIA, Rater-1 and Rater-2 is shown in Fig. 1. Note that the segmentation result of BraTumIA indicates the same volume progression as the manual segmentations. From a total of 64 MR acquisitions, tumor volumes were obtained and corresponding volume differences (*n* = 50) computed. In Fig. 2 absolute volumes and volume differences between consecutive time points as measured by BraTumIA were plotted against the estimates of Rater-1 and Rater-2. Strong significant correlations (r-values ranging from 0.83 to 0.96, *p* < 0.001) were observed between all estimates of BraTumIA and of each of the raters. The measured Dice-coefficients between BraTumIA and each of the human raters are reported in Table 2.

### Segmentation Tendencies

**Figure 3:**
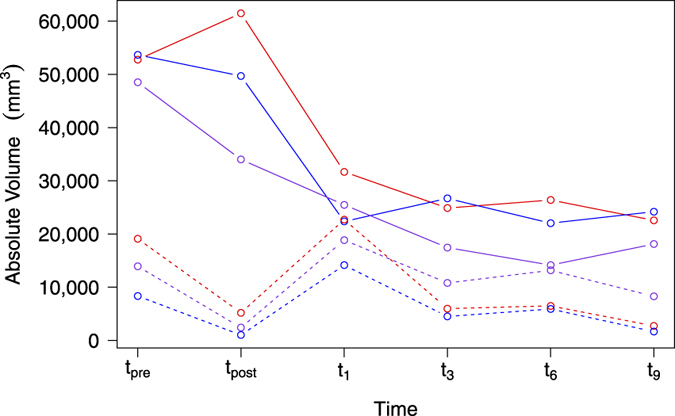
Volumetric evolution of non-enhancing *T*_2_-hyperintense tissue (NCE-*T*_2_, solid line) and contrast-enhancing tumor (CET, dashed line) over time for BraTumIA (red), Rater-1 (blue) and Rater-2 (violet) shown for one patient.

In Fig. 3 the volumetric trend lines of a representative patient as defined by the different raters are shown. For the NCE-*T*_2_ compartment, BraTumIA tended to yield a larger postoperative absolute volume than the human raters. For the contrast-enhancing tumor, one can note a larger absolute volume in case of the preoperative, the immediate postoperative measurement and one month follow-up, whereas for the remaining follow-up images the volumetric estimates of BraTumIA were located between the estimates of the human raters. When computing the relative over- or underestimation of BraTumIA with respect to each human rater and of all patients, a similar trend as for the patient shown in Fig. 3 can be observed. Table 3 gives an overview of the tendencies of BraTumIA with respect to the human raters. A consistent overestimation for all postoperative time points in the case of the NCE-*T*_2_ compartment as well as for the preoperative and immediate postoperative CET volumes was observed. For the segmentation of CET in the remaining follow-up images BraTumIA yielded absolute volumes within the range of volumes defined by both human raters.

### Longitudinal Volumetry

**Figure 4:**
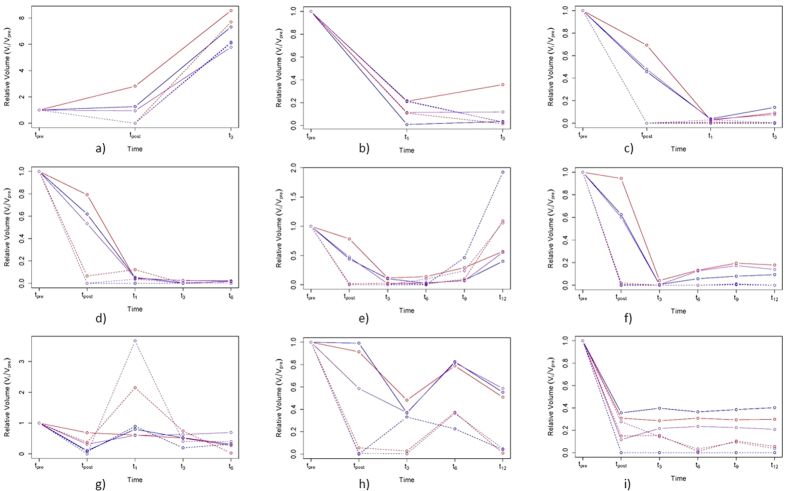
Volumetric evolution of relative values with respect to preoperative volume of non-enhancing *T*_2_-hyperintense tissue (NCE-*T*_2_, solid line) and contrast-enhancing tumor (CET, dashed) over time for BraTumIA (red), Rater-1 (blue) and Rater-2 (violet). The figures “a” to “i” are different patients.

The volumetric estimates for the NCE-*T*_2_ compartment and CET were normalised with respect to the first measurement (i.e. *t*_*pre*_). This way, the individual tendencies shown before can be mitigated and differences in the volumetric trend between raters can be pointed out more easily. In Fig. 4, the relative volumetric curves for nine patients are shown. For the first six patients, a general agreement between all three raters on the trend of the NCE-*T*_2_ compartment as well as contrast-enhancing tumor was observed. For patient “g”, the occurrence of a lamellar enhancement close to the resection site of the primary tumor led to a higher increase in CET volume computed by BraTumIA and Rater-2 compared to Rater-1. For patient “h”, a diffuse and weak tumor enhancement occurred, which was only detected by Rater-1 (at *t*_3_). For patient “i”, we can observe disagreements between all three raters for small NCE-*T*_2_ volume changes occurring after surgery. From a qualitative point of view, 11 out of 14 patients showed a good agreement among all three raters. Three patients (two of them depicted in Fig. 4) showed a (clinically) significant deviation between the raters, whereas one case (depicted in supplementary Fig. S1 “a”) was a patient undergoing biopsy instead of surgical resection. A complete overview of the longitudinal volumetric curves for all 14 patients is given by Figs 4 and S1.

### Inter-rater Disagreement

**Figure 5:**
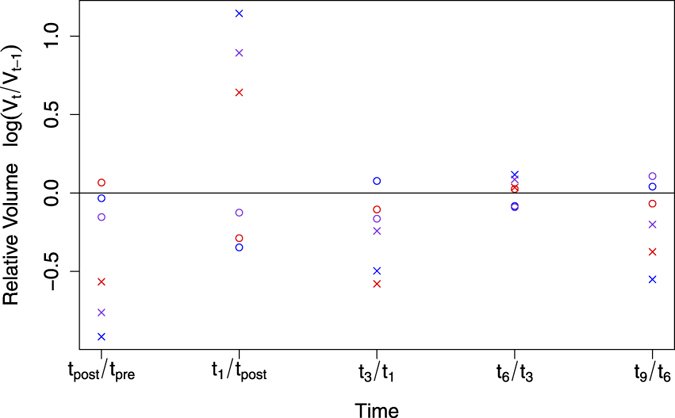
Disagreement plot for the patient shown in Fig. 3. Measurements for non-enhancing *T*_2_-hyperintense tissue (NCE-*T*_2_) are indicated with circles, for contrast-enhancing tumor (CET) with crosses. Estimates of Bratumia are shown in red, of Rater-1 in blue and of Rater-2 in violet. The logarithm is used to facilitate visualisation. The zero line indicates a stable volume (i.e. Δ_*rel*_ = 1) from one time point to the next one. A positive value indicates a volume increase, whereas a negative value indicates a volume decrease between consecutive time points. Estimates of two raters which lie on different sides of the zero line are regarded as a disagreement between the two raters.

**Figure 6:**
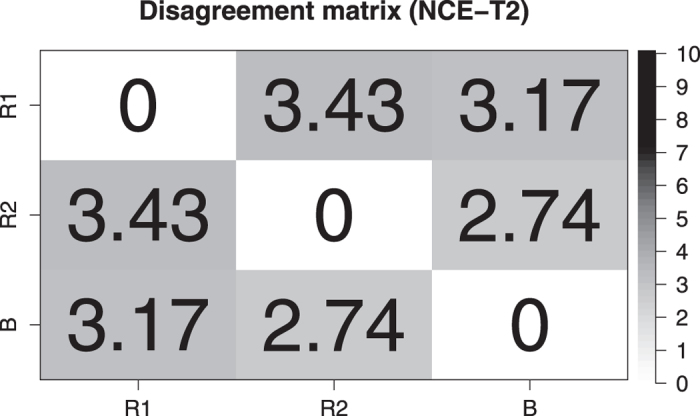
Disagreement matrix for non-enhancing *T*_2_-hyperintense tissue (NCE-*T*_2_). Each entry reflects the total amount of disagreement between two raters. A disagreement is measured as the absolute difference between the estimates of two raters. It is measured if the estimates lie on different sides of the zero line in the disagreement plot (Fig. 5). The total amount of disagreement is the sum of disagreements over all patients.

**Figure 7:**
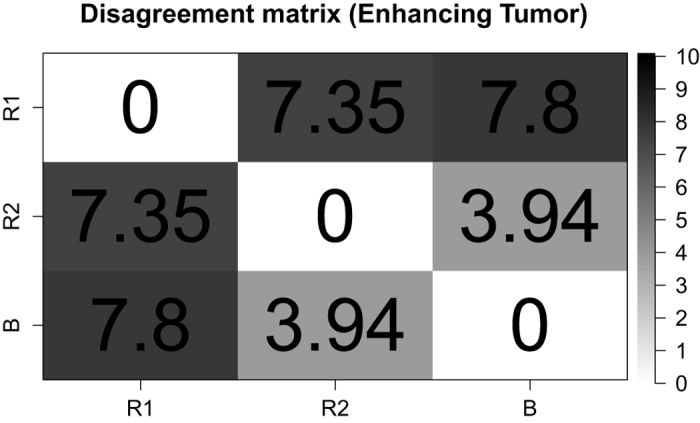
Disagreement matrix for contrast-enhancing tumor (CET). Each entry reflects the total amount of disagreement between two raters. A disagreement is measured as the absolute difference between the estimates of two raters. It is measured if the estimates lie on different sides of the zero line in the disagreement plot (Fig. 5). The total amount of disagreement is the sum of disagreements over all patients.

In total, there are 64 individual time points wherein a segmentation was performed. This yielded a total of 50 transitions from one time point to the next, where potentially a disagreement can occur. When comparing the trend lines of BraTumIA against Rater-1 for all 50 transitions, a disagreement for the trend of the NCE-*T*_2_ compartment was found in 11 transitions (22%) and in four transitions (8%) for the contrast-enhancing tumor. When comparing BraTumIA against Rater-2, a disagreement was detected for the NCE-*T*_2_ compartment in 12 occurrences (24%), and in two transitions (4%) for the contrast-enhancing tumor. For the two human raters, a disagreement for the NCE-*T*_2_ compartment occurred in 13 transitions (26%) and in four transitions (8%) for the contrast-enhancing tumor. An agreement on the trend line between both human raters but a disagreement of BraTumIA was found in six transitions out of 50 (12%) for the NCE-*T*_2_ tissue (three of which are located in the patient shown in Fig. 3), however, did not occur for the contrast-enhancing tumor. The disagreement plot for an exemplary patient is shown in Fig. 5. Figures 6 and 7 present the disagreement matrices for NCE-*T*_2_ tissue and CET, respectively. When comparing the two matrices, one can observe that if a disagreement between raters (as defined in section 2.5) occurs, it is larger for the contrast-enhancing volume than for the NCE-*T*_2_ tissue. In general, BraTumIA disagreed less with Rater-2 than with Rater-1. For the NCE-*T*_2_ compartment the total disagreement between BraTumIA and either one of the two human raters (3.17 and 2.74) was smaller than the disagreement between the two human raters (3.43). For the contrast-enhancing tumor the total disagreement between BraTumIA and Rater-1 (7.8) was larger than the disagreement between the two human raters (7.35).

## Discussion

The study at hand provides evidence for the capability of a fully-automatic segmentation method for longitudinal brain tumor volumetry via comparison of its performance against two expert raters. Brain tumor segmentation is routinely needed in radiation oncology, where it plays a crucial role in the planning of radiotherapy. The output of a tumor segmentation yields information about its volume as well as its position relative to neighboring and potentially eloquent anatomical structures. Manual segmentation is inherently subjective, thus the estimated volumes show large variability when compared between different raters^8^. In radiotherapy, the planning is greatly influenced by an accurate estimation of the target volumes, which means that segmentations of different raters can lead to different treatment plans. Early on, researchers in radiation oncology investigated computer-assisted methods to relieve clinicians from the time-consuming burden of manual segmentation as well as generate more consistent volumetric estimates^32^. The potential areas of applications for brain tumor volumetry span well beyond radiotherapy and include neurooncology (response assessment^6^,^27^,^33^,^34^,^35^,^36^), neurosurgery^37^,^38^,^39^ and radiogenomics^12^,^40^,^41^.

BraTumIA is a fully-automatic, machine learning-based segmentation model capable of subdividing a glioma into its compartments (necrosis, edema, enhancing and non-enhancing tumor). The performance of BraTumIA was compared against alternative approaches in the MICCAI BraTS Challenges^10^,^17^, where it proved to be one of the best performing as well as fastest methods. Moreover, BraTumIA was evaluated prospectively on clinical data sets for preoperative brain tumor segmentation in the past^19^. Strong correlations were observed between automatically and manually generated tumor volumes. Furthermore, the estimated tumor volumes of BraTumIA were recently shown to be associated with patient survival^42^. The software is equipped with a Graphical User Interface (GUI), which facilitates its use by clinicians. In addition, it has been made publicly-available (https://www.nitrc.org/projects/bratumia/). This led us to the decision to employ BraTumIA for performing automatic longitudinal tumor volumetry.

The agreement between the segmentation result of BraTumIA and the estimates of the two human raters was assessed via computing Dice-coefficients. Looking at Table 2, it is evident that the preoperative segmentation task seems to be the easiest both for BraTumIA and the human raters. Notice that the measured preoperative Dice-coefficients are comparable to the values reported during the MICCAI BraTS 2012/2013 challenges^10^. Furthermore, the segmentation of residual CET after surgery is extremely challenging, which is confirmed by the low Dice-coefficients for all pairings of raters. However, overlap measures such as the Dice-coefficient are overly sensitive if the segmented image regions are small in size. This is the case for residual CET in postoperative images. The segmentation of both NCE-*T*_2_ tissue and CET appears to be challenging in the one month follow-up image acquisition. The reason is the emergence of changes in image appearance at that time similar to pathological changes (e.g. benign contrast-enhancement) but in fact they are induced by the treatment. A reason for the decrease in volumetric overlap between the estimates of BraTumIA and the two human raters for postoperative images could be the fact that a major part of the available training data (i.e. 36 data sets) were preoperative images. The addition of more postoperative images would likely improve the performance of BraTumIA for segmenting later time points. A thorough comparison of the segmentation performance of BraTumIA to several other segmentation techniques is provided by Menze *et al*.^10^ based on the results of the MICCAI BraTS challenges 2012/2013 (in this publication BraTumIA is referred to as the approach proposed by Meier *et al*.^17^).

BraTumIA proved to effectively capture the volumetric trend of the NCE-*T*_2_ compartment and CET over time. A trend towards overestimation after surgery with respect to both human raters for the NCE-*T*_2_ tissue was observed. This corresponds to a relatively stable bias of the volumetric trend curve as generated by BraTumIA. In neurooncology, criteria for assessing response to therapy rely on relative changes in tumor size between a given point in time and a baseline^27^. Thus, a decision on response to therapy is not affected by a constant bias of the chosen measurement method. For the CET volume measured immediately after surgery, BraTumIA tends to oversegment the residual tumor volume and in case of complete resection rarely segmented a volume of zero *mm*^3^. Thus, the high values reported in Table 3 for immediate postoperative CET volumes are due to the fact that seven out of 14 patients underwent complete resection. Residual tumor volumes are usually small and often rated as unmeasurable by the current response assessment criteria. In order to employ BraTumIA for response assessment, it is crucial to study the effect of changing non-measurable lesions to measurable lesions. This effect may be emphasised due to the overestimation of residual volumes when compared with manual volumetry. More specialised algorithms developed specifically for the assessment of residual tumor volume after surgery have been proposed^7^,^43^,^44^ and may yield an improved performance over general segmentation methods like BraTumIA.

Our results show that the longitudinal volumetry of the CET generated by BraTumIA is closer to Rater-2 than Rater-1. In general, BraTumIA as well as Rater-2 tend to segment CET more aggressively than Rater-1. This is clearly visible in their oversegmentation of CET compared to Rater-1. This can potentially lead to false positives as for example in case of patient “g”, where both BraTumIA and Rater-2 marked a lamellar enhancement appearing at the border of the resection cavity (one month after surgery) as contrast-enhancing tumor. Follow-up images revealed that the enhancement was a consequence of the applied treatment regime. However, for patient “h” BraTumIA and Rater-2 failed to recognise a subtle contrast-enhancing tumor (at *t*_3_). Rater-1 was an experienced neuroradiologist, whereas Rater-2 was a M.D. student with initial experience in the field. Consequently, one can argue that Rater-1 relied in both situations on his experience as well as knowledge about the patient’s history and applied treatment regime to either rule in or rule out the presence of contrast-enhancing tumor. However, BraTumIA is segmenting every patient image individually and does not include information extracted from previous scans of the same patient. Hence, we think that a possibility for improving BraTumIA might be a more explicit inclusion of past imaging information of a patient^45^ as well as information from clinical variables about the applied treatment regime (e.g. radiotherapy yes/no). This may enable BraTumIA to better rule out non-tumorous contrast-enhancements as well as to detect subtle changes within a patient. Furthermore, we observed that for the NCE-*T*_2_ compartments, disagreements occurred predominantly for small relative changes in volume (e.g. for patient “i”). In the context of response assessment, such changes would likely be rated as stable disease.

The analysis of disagreements between raters was driven by the importance of the relative change in tumor size for tumor response assessment^1^,^27^. BraTumIA showed a disagreement with two expert raters that lied in the range of the disagreement between the human raters themselves. Bi-dimensional measures used for response assessment have well known limitations. Wen *et al*.^27^ suggested that a main obstacle for the inclusion of volumetric information in response assessment is the lack of standardisation in volumetric imaging. In addition, the necessary time to manually acquire volumetric information renders its clinical use unfeasible. BraTumIA was trained on imaging data that is not part of this study and which was acquired following different acquisition protocols. The software is capable of generalising imaging patterns of CET and NCE-*T*_2_ compartments learned from training data to unseen patient data. Moreover, BraTumIA can provide volumetric information fully-automatically for tumor compartments within five minutes (average computation time per patient).

We proposed disagreement plots and matrices for evaluating the disagreement between different raters. An alternative method to analyse the disagreement between raters or measurement methods is the Bland-Altman plot^46^, which is widely used (e.g. by Bauknecht *et al*.^47^). The main goal of our data analysis was to capture and quantify deviations between the volumetric trend for CET and the NCE-*T*_2_ compartment estimated by BraTumIA and the two raters. A Bland-Altman approach for this issue would require one plot for every transition between consecutive time points and every possible pairing of measurement methods or raters. Given the small sample size for late postoperative transitions (e.g *n* = 4 from *t*_6_ to *t*_9_), the estimation of 95% limits of agreement in the Bland-Altman plot would be unreliable. Consequently, we opted for a more descriptive and visual approach in form of disagreement plots and matrices. A disagreement plot is patient-specific and retains the complete temporal evolution as well as the entirety of the employed raters. In a next step, a specific feature (e.g. the absolute differences between disagreements) of the data can then be summarised among all patients, e.g. in the form of disagreement matrices. Different definitions of disagreements would result in different matrices that may not necessarily be symmetric. For instance, for a larger number of raters, the correlation between disagreement matrices could potentially be assessed by a Mantel test^48^. Weizman *et al*.^21^ followed a different approach for assessing the agreement between longitudinal volumetric curves. They estimated Pearson’s correlation coefficients between two subsequent measurements of two raters. This requires the assumption of a linear relationship between the volumetric measurement of two consecutive time points. Furthermore, summarising correlation coefficients over a number of samples (=patients) is not straightforward. In contrast, the use of disagreement plots and matrices is nonparametric and allows the extraction of different features, which can be summarised over all patients.

There are two limitations of the present study. First, we limited our analysis to the two morphologically most discriminative tumor compartments. This restriction was driven by the lack of histologically-confirmed ground truth data for postoperative images. However, in clinical practice a radiologist segments the extent of the tumor compartment that is visually apparent in MR images. In light of the fact that the postoperative discrimination between edema and non-enhancing tumor is very difficult even for expert raters and due to the absence of its histological confirmation, we combined the segmentation of edema and non-enhancing tumor in one compartment (i.e. the non-enhancing *T*_2_-hyperintense tissue). Furthermore, the chosen approach of analysing the non-enhancing *T*_2_-hyperintense part of the tumor was used previously^20^,^40^. Second, a higher number of human raters would have allowed us to conduct a more informative assessment of the inter-rater variability of manual segmentations when compared with the automatic segmentations generated by BraTumIA. The methodology of employing two human raters for segmentation has been used in several studies before, as reported by Bauknecht *et al*.^47^.

In summary, BraTumIA generated volumetric trend curves of contrast-enhancing and non-enhancing *T*_2_-hyperintense tumor compartments comparable to estimates of human raters. Strong correlations between the volumetric estimates of BraTumIA and the two raters were observed. The frequency and extent of disagreement between BraTumIA and either one of the two human raters was comparable to the values measured between the two human raters themselves. This implies that BraTumIA can be used as a substitute for manual volumetric follow-up of contrast-enhancing and non-enhancing *T*_2_-hyperintense tumor compartments over time.

## Additional Information

**How to cite this article**: Meier, R. *et al*. Clinical Evaluation of a Fully-automatic Segmentation Method for Longitudinal Brain Tumor Volumetry. *Sci. Rep.* **6**, 23376; doi: 10.1038/srep23376 (2016).

## Supplementary Material

Supplementary Information

## Figures and Tables

**Table 1:** Longitudinal patient data used for evaluation.

Patient	*t*_*pre*_	*t*_*post*_	*t*_1_	*t*_3_	*t*_6_	*t*_9_	*t*_12_	Total:
4 a)	✓	✓	–	✓	–	–	–	3(2)
4 b)	✓	–	✓	✓	–	–	–	3(2)
4 c)	✓	✓	✓	✓	–	–	–	4(3)
4 d)	✓	✓	✓	✓	✓	–	–	5(4)
4 e)	✓	✓	–	✓	✓	✓	✓	6(5)
4 f)	✓	✓	–	✓	✓	✓	✓	6(5)
4 g)	✓	✓	✓	✓	✓	–	–	5(4)
4 h)	✓	✓	–	✓	✓	–	✓	5(4)
4 i)	✓	✓	–	✓	✓	✓	✓	6(5)
S1 a)	✓	–	✓	✓	–	–	–	3(2)
S1 b)	✓	✓	✓	–	–	–	–	3(2)
S1 c)	✓	✓	✓	✓	–	–	–	4(3)
S1 d)	✓	✓	✓	✓	✓	–	–	5(4)
S1 e)	✓	✓	✓	✓	✓	✓	–	6(5)
Total:	14	12	9	13	8	4	4	64(50)

Patients are referred to via figure numbers. The check mark indicates if a particular Magnetic Resonance (MR) acquisition was performed for a given patient. Every patient has a preoperative MR acquisition (*t*_*pre*_) and several postoperative MR acquisitions, where *t*_*post*_ refers to the immediate postoperative scan and the index *i* in *t*_*i*_ refers to the month after surgery in which the MR acquisition was performed. There is a total of 64 MR acquisitions yielding 50 time differences between subsequent scans (shown in parentheses). Notice that this data is independent from the data used for training, which is described in subsection “Fully-automatic Segmentation”.

**Table 2:** Dice-coefficients for non-enhancing *T*_2_-hyperintense tissue (NCE-*T*_2_) and contrast-enhancing tumor (CET) as tuple (median, range) generated by BraTumIA (B) with respect to either one of two human raters (R1/R2).

Comparison	*t*_*pre*_(*n* = 14)	*t*_*post*_(*n* = 12)	*t*_1_(*n* = 9)	*t*_3_(*n* = 13)	*t*_6_(*n* = 8)	*t*_9_(*n* = 4)	*t*_12_(*n* = 4)
B vs. R1 (NCE-*T*_2_)	(0.746, 0.319)	(0.673, 0.716)	(0.408, 0.778)	(0.589, 0.803)	(0.467, 0.549)	(0.586, 0.573)	(0.729, 0.246)
B vs. R2 (NCE-*T*_2_)	(0.739, 0.376)	(0.633, 0.484)	(0.424, 0.551)	(0.592, 0.815)	(0.495, 0.483)	(0.508, 0.571)	(0.71, 0.166)
R1 vs. R2 (NCE-*T*_2_)	(0.792, 0.45)	(0.747, 0.657)	(0.596, 0.731)	(0.652, 0.701)	(0.603, 0.483)	(0.619, 0.258)	(0.787, 0.389)
B vs. R1 (CET)	(0.661, 0.647)	(0.183, 0.349)	(0.398, 0.489)	(0.205, 0.746)	(0.419, 0.589)	(0.579, 0.224)	(0.435, 0.779)
B vs. R2 (CET)	(0.687, 0.69)	(0.239, 0.347)	(0.35, 0.605)	(0.279, 0.827)	(0.295, 0.732)	(0.433, 0.222)	(0.403, 0.695)
R1 vs. R2 (CET)	(0.74, 0.34)	(0.116, 0.345)	(0.464, 0.454)	(0.551, 0.549)	(0.502, 0.426)	(0.567, 0.417)	(0.752, 0.176)

Number of patients having a particular acquisition is denoted by *n*.

**Table 3:** Relative over- or underestimation of the volumes for non-enhancing *T*_2_-hyperintense tissue (NCE-*T*_2_) and contrast-enhancing tumor (CET) as median values generated by BraTumIA (B) with respect to either one of two human raters (R1/R2).

Comparison	*t*_*pre*_(*n* = 14)	*t*_*post*_(*n* = 12)	*t*_1_(*n* = 9)	*t*_3_(*n* = 13)	*t*_6_(*n* = 8)	*t*_9_(*n* = 4)	*t*_12_(*n* = 4)
B vs. R1 (NCE-*T*_2_)	0.969	1.528	1.27	1.188	1.71	1.539	1.098
B vs. R2 (NCE-*T*_2_)	1.023	1.763	1.243	1.282	1.103	1.194	1.142
R1 vs. R2 (NCE-*T*_2_)	1.011	1.077	0.941	1.018	0.845	1.27	0.944
B vs. R1 (CET)	1.357	9.904	1.605	1.326	1.429	1.344	1.123
B vs. R2 (CET)	1.055	6.578	0.726	0.882	0.545	0.520	0.979
R1 vs. R2 (CET)	0.719	1	0.495	0.765	0.646	0.363	0.926

Number of patients having a particular acquisition is denoted by *n*.
