# Supplementary material for: Clinical Evaluation of a Fully-automatic Segmentation Method for Longitudinal Brain Tumor Volumetry
Source: Sci Rep. 2016 Mar 22;6:23376. doi: 10.1038/srep23376 (PMC4802217; doi:10.1038/srep23376)
Supplement: Supplementary Information [file srep23376-s1.pdf]

Clinical Evaluation of a Fully-automatic Segmentation Method for Longitudinal Brain Tumor Volumetry

Raphael Meier[1,+,*], Urspeter Knecht[2,+], Tina Loosli[2], Stefan Bauer[1,2], Johannes Slotboom[2], Roland Wiest[2,†], and Mauricio Reyes[1,†,*]

[1]Institute for Surgical Technology & Biomechanics, University of Bern, Bern, Switzerland
[2]Support Center for Advanced Neuroimaging – Institute for Diagnostic and Interventional Neuroradiology, University Hospital and University of Bern, Bern, Switzerland
[*]raphael.meier@istb.unibe.ch, mauricio.reyes@istb.unibe.ch
[+]these authors share first authorship
[†]these authors share senior authorship

ABSTRACT

This document contains **supplementary material**.

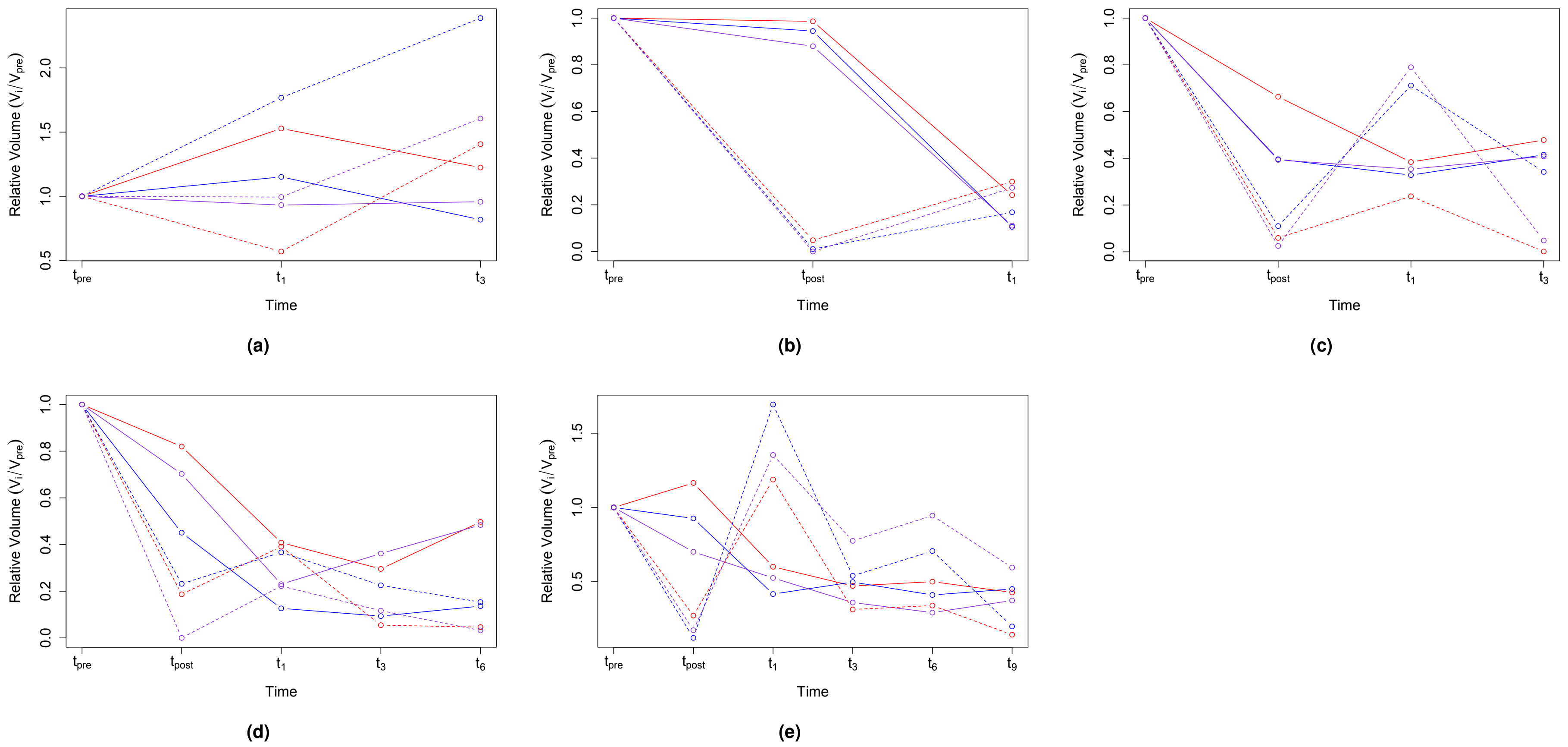

Supplementary Figure S1. Volumetric evolution for the remaining five of 14 patients. The relative values with respect to preoperative volume of non-enhancing T_2-hyperintense tissue (NCE-T_2, solid line) and contrast-enhancing tumor (CET, dashed) over time for BraTumIA (red), Rater-1 (blue) and Rater-2 (violet) are shown. The figures "a" to "e" are different patients.
